# Microstructure and Magnetic Properties of Nanocrystalline Fe_60−x_Co_25_Ni_15_Si_x_ Alloy Elaborated by High-Energy Mechanical Milling

**DOI:** 10.3390/ma15186483

**Published:** 2022-09-19

**Authors:** Nawel Khitouni, Béchir Hammami, Núria Llorca-Isern, Wael Ben Mbarek, Joan-Josep Suñol, Mohamed Khitouni

**Affiliations:** 1 Laboratory of Inorganic Chemistry (LR17ES07), Faculty of Sciences of Sfax, University of Sfax, Sfax 3029, Tunisia; 2 Department of Physics, University of Girona, Campus Montilivi, 17071 Girona, Spain; 3 Department of Chemistry, Qassim University, Buraidah 51452, Saudi Arabia; 4 Department of CM-QF, Universitat de Barcelona, Martí Franquès 1, 08028 Barcelona, Spain

**Keywords:** mechanical alloying, Fe-Co-Ni-Si alloy, magnetic properties

## Abstract

In the present work, the effect of Si addition on the magnetic properties of Fe_60−x_Co_25_Ni_15_Si_x_ (x = 0, 5, 10, 20, and 30 at%) alloys prepared by mechanical alloying was analyzed by X-ray diffraction and magnetic vibrating sample magnetometry and SQUID. The crystallographic parameters of the bcc-solid solutions were calculated by Rietveld refinement of the X-ray diffraction patterns with Maud software. Scanning electron microscopy (SEM) was used to determine the morphology of the powdered alloys as a function of milling time. It was found that the Si addition has an important role in the increase of structural hardening and brittleness of the particles (favoring the more pronounced refinement of crystallites). The resulting nanostructure is highlighted in accordance with the concept of the structure of defects. Magnetic properties were related to the metalloid addition, formed phases, and chemical compositions. All processed samples showed a soft ferromagnetic behavior (Hc ≤ 100 Oe). The inhomogeneous evolution of the magnetization saturation as a function of milling time is explained by the magnetostriction effective anisotropy and stress induced during mechanical alloying.

## 1. Introduction

Fe–Co-based alloys are of great interest because they have good soft magnetic behavior such as high specific saturation magnetization, high permeability, and elevated Curie temperature that make them attractive for high-temperature applications and suitable for magnetic recording and magnetic fluids [1,2,3,4]. Fe–Ni alloys are promising candidates for wide applications in technology since they exhibit good soft properties but have low resistivity [5,6,7]. In recent years, there is a great demand for soft magnetic materials such as steels with high carbon or silicon which are brittle at room temperature. However, it has been found that adding Ni to Fe–Co materials improves permeability and resistivity at the same time [8]. As a result, the Fe–Ni–Co alloys are required for applications as soft magnetic materials because of their higher saturation magnetization (Ms), lower coercivity (Hc), and high resistivity. Moreover, the addition of a certain amount of B, C, P, and Si to α-Fe-based alloys has been shown to promote thermal and soft magnetic properties [9,10]. Mechanical alloying (MA) is an interesting technique used to prepare amorphous or nanostructured magnetic alloys and even to produce metastable phases of immiscible systems. Significant changes in the microstructure during the high-energy mechanical alloying can greatly affect the magnetic properties [11]. The thermal relaxation of the stress that occurred during the milling process can cause an increase of saturation magnetization (Ms) and a decrease of coercivity (Hc). It has been reported that the magnetic properties of an alloy are related to the basic metal mixture, the alloying elements, and the crystal structures [12,13,14,15,16,17]. Indeed, saturation magnetization depends on the compositional and structural changes, and resulting phase constitution [18], while Hc is linked to the grain size, the defects density, impurities, and lattice strains [19]. Research works have investigated the important role of microstructure and phase transformation, that occurred during milling, on the changes in the magnetic properties of Fe-based alloys [18,19,20,21,22,23,24] and studied their restricted applications. The investigation of structural defects’ role in nanostructure and magnetic behaviors seems useful for understanding their correlation with microstructure and phase transformations in Fe-based alloys. The crystalline defects introduced in terms of dislocations, grain boundaries, stacking defects, etc., during high-energy milling must be mentioned as the main causes for the enhancement of the magnetic properties of these compounds. For that, the comprehension of the relationship structure defects’ magnetic behavior could be considered among the current topics of research. The main objective of this work can be briefly explained as follows: we presented a mechanosynthesis of ternary FeCoNi doped with Si for the comprehension of the Si addition’s influence on the microstructure and magnetic behavior. The representation of the variation of the magnetic parameters according to those of the microstructure may facilitate the comprehension of the important role of structural defects in good magnetic properties. In this work, the compositions Fe_60−x_Co_25_Ni_15_Si_x_ (x = 0, 5, 10, 20, and 30 at.%) were synthesized by high-energy mechanical alloying to find out the effect of both the Si addition and the structural defects in the magnetic behavior of the alloy.

## 2. Materials and Methods

The elemental powders of Fe (99.97% purity, mean particle size < 10 μm), Co (99.9% purity, mean particle size < 2–5 μm), Ni (99.7% purity, mean particle size < 10 μm), and Si (99.5% purity, mean particle size < 10 μm) were mechanically alloyed in an Ar atmosphere to synthesize the Fe_60−x_Co_25_Ni_15_Si_x_ (x = 0, 5, 10, 20, and 30) (at.%) powders by using a planetary ball mill (Fritsch P7). The ball-to-powder weight ratio was chosen to be 2:1, and the milling intensity was set at 600 rpm. The milling was handled up to 100 h with a sequence consisting of 10 min of milling followed by 5 min of an idle period, to prevent the powder from sticking to the walls of the container and the balls and its agglomeration during milling. X-ray diffraction (XRD) patterns were collected using a D-500 Siemens equipment with CuKα radiation (λ = 0.15406 nm). The microstructural parameters such as crystallites size, lattice strains, and lattice parameter were obtained by Rietveld refinement using the Maud program [25]. In all refined XRD patterns, quality refinement parameter R_exp_ is lower than 10.5% and GOF parameter lower than 1.6. The morphology and the composition of MA powders were determined by scanning electron microscopy (SEM) in a DSM960A ZEISS microscope in secondary electron mode operating at a voltage of 15 kV. The SEM was equipped with a Vega-Tescan energy dispersive X-ray spectrometry (EDS) analyzer. The magnetic characterization was carried out by Superconducting Quantum Interference Device from Quantum Design SQUID MPMS-XL at 300 K.

## 3. Results and Discussion

### 3.1. SEM Analysis

**Figure 1:**
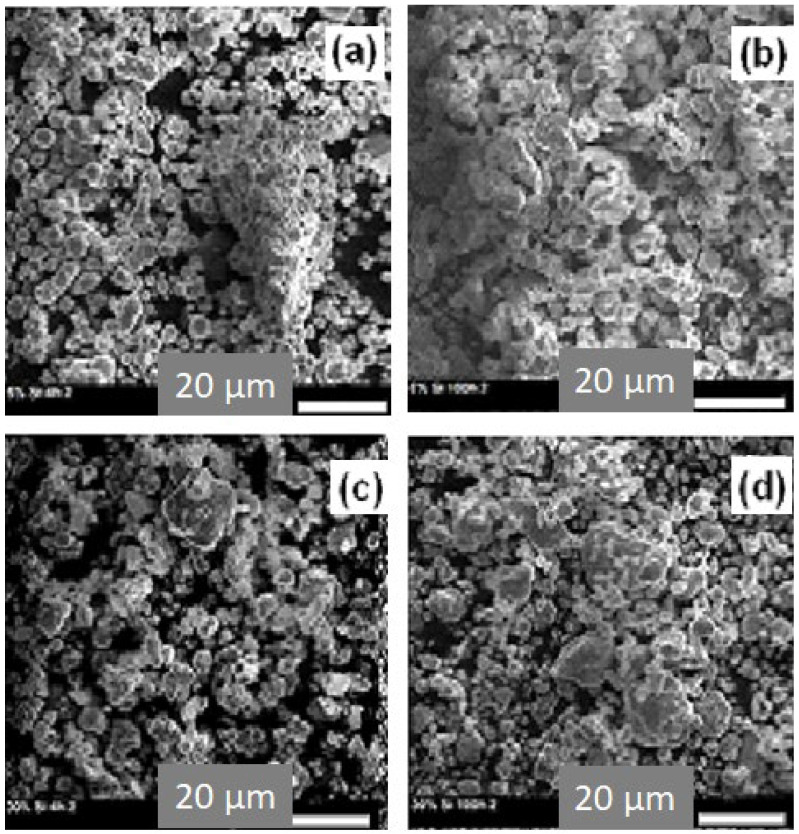
SEM micrographs for powders: Fe_55_Co_25_Ni_15_Si_5_ obtained after 4 h (**a**) and after 100 h of milling (**b**) and Fe_30_Co_25_Ni_15_Si_30_ milled for 4 h (**c**) and for 100 h (**d**), respectively.

Figure 1 gives typical examples of the SEM images of the alloyed Fe_55_Co_25_Ni_15_Si_5_ and Fe_30_Co_25_Ni_15_Si_30_ powder mixtures obtained after 4 and 100 h of milling. The particle size was reduced from around 7 μm in both samples with increased milling time up to 100 h. Two deformation mechanisms are responsible for the final microstructure: plastic deformation (associated with cold welding in ductile powders and fracture in hard powders) [26,27]. Indeed, the Si particles are harder and more brittle than iron, cobalt, and nickel particles, so they are fractured and the small ones have consolidated to the lamellar particles, and then agglomerated particles with different sizes and shapes are formed [28]. Figure 1a,c shows the morphology of the 4 h powders; the particles appear fine (~7 µm) and less agglomerated. This state can be related to the compressive forces introduced into the deformed particles after the successive ball–powder–ball collisions show that for all compositions and powders, larger particles with irregular shapes and sizes are formed due to cold welding. After an increase in milling up to 100 h, particles appear in spherical shapes and more uniform size distribution, as a result of intensive fracture after a preliminary cold-welding stage. This is due to the dissolution of Si in the metallic matrix indicating that the alloying process will take place (Figure 1b,d). Moreover, an increase in milling time leads to a work hardening (due to mechanically induced crystallographic defects) of the particles, and then fracturing occurs. In addition to the milling time, the competition between fracturing and welding processes under the effect of severe plastic deformation depends on the silicon content. In fact, at low content, metallic particles are soft and malleable and tend to weld together and form an agglomerate particle (larger particles), whereas for high Si content, the particles get work-hardened and tend to fracture, and the size of the particles diminishes (Figure 1b,d).

**Figure 2:**
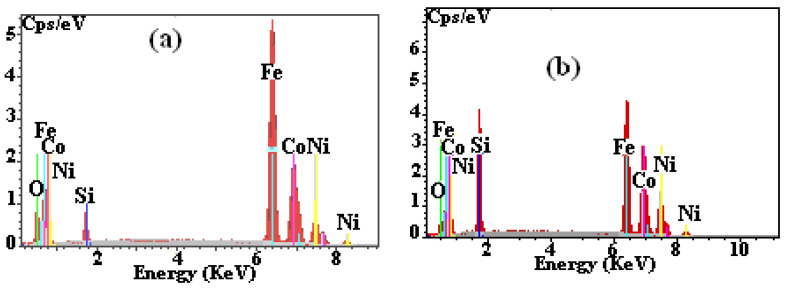
EDS spectra of milled (100 h) powdered alloys: Fe_55_Co_25_Ni_15_Si_5_ (**a**) and Fe_30_Co_25_Ni_15_Si_30_ (**b**).

Figure 2 shows the EDS analysis of the powder mixtures containing 5 and 30% Si milled for 100 h, respectively. Microanalysis confirms that the percentage ratio of the original elements is very close to the nominal values in both cases (56.35at%Fe-24.1at%Co-14.8at%Ni-4.75at%Si and 31.48at%Fe-25.1at%Co-14.3at%Ni-29.12at%Si). Nevertheless, EDS also reveals the existence of some traces of oxygen as well as the four mixture elements. This may be the cause of contact of the milled powder with the oxygen in the air during its collection from jars.

### 3.2. XRD Analysis

**Figure 3:**
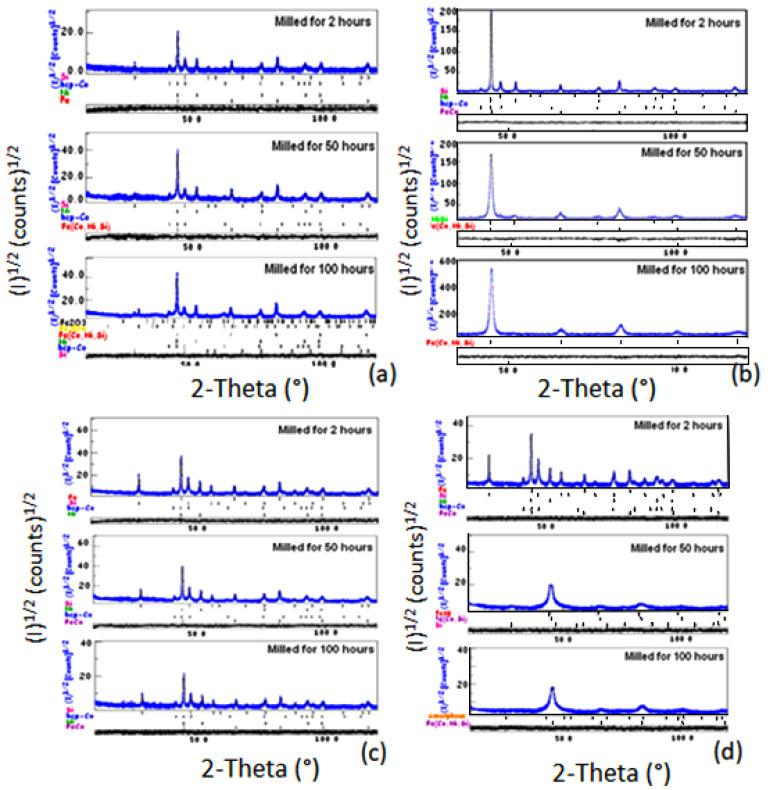
XRD diffractograms (experimental, refinement) of powdered mixtures containing 5 (**a**), 10 (**b**), 20 (**c**), and 30 (**d**) at.% Si. Alloys are milled for 2, 50, and 100 h.

The structure and phases evolution of Fe_60−x_Co_25_Ni_15_Si_x_ powders (x = 0, 5, 10, 20 and 30 at.%) were examined according to the milling time. The Rietveld analysis of the XRD patterns of alloys containing 5, 10, 20, and 30 at.% of Si, obtained after 2, 50, and 100 h of milling, are shown in Figure 3. The goodness of fit (GOF) refinement parameter was always <1.23.

The refinement of the XRD diffractograms of the powder with 5%Si, milled for 2 h, identifies the following phases: basic bcc-iron (Im-3m; a = 2.8665(1) Å; wt(%)~55), hcp-cobalt (P63/mmc; a = 2.5057(1) Å and c = 4.0713(1) Å; wt(%)~25), fcc-nickel (Fm-3m; a = 3.5245(1) Å; wt(%)~15), and cubic-silicon (Fd-3m; a = 5.4355(2)Å; wt(%)~5) (Figure 3a). Prolonging the milling time up to 50 h, we remark the appearance of the bcc-Fe(Co,Ni,Si) supersaturated solid solution with lattice parameters 2.8661(1) Å and phase proportion around 51%. After 100 h milling, the final milling product is multiphase where the refined phases are the bcc-Fe(Co,Ni,Si) (Im-3m; a = 2.8663(1)Å; wt(%)~62) and some proportions of the hcp-cobalt (P63/mmc; a = 2.5040(1)Å and c= 4.0684(1) Å; wt(%)~7), fcc-nickel(Fm-3m; a = 3.5239(1) Å; wt(%)~7), and cubic-silicon (Fd-3m; a = 5.4322(2) Å; wt(%)~6) which still remain free. At the same time, we refined some traces of iron oxides in the forms Fe_2_O_3_ (R-3C; a = 5.0355(2) and c = 13.7471(1) Å; wt(%)~8) and Fe_3_O_4_ (Fd-3m; a = 8.3967(2) Å; wt(%)~10). In the case of the mixture of powders containing 10% Si, the Rietveld refinement confirms the formation of the solid solution bcc-FeCo (Im-3m; a = 2.8687(1) Å; wt(%)~20) after 2 h, while after 50 h, Co as a free element disappears completely by entering into the Fe lattice to form the bcc-Fe(Co,Ni,Si) (Im-3m; a = 2.8623(1) Å; wt(%)~88) supersaturated solid solution with a small proportion of the fcc-Ni(Si) (Fm-3m; a = 3.5337(1) Ǻ; wt(%)~12) (Figure 3b). After 100 h of milling, we identify only the phase bcc-Fe(Co,Ni,Si) (Im-3m; a = 2.8563(1) Å; wt(%)~100).

**Figure 4:**
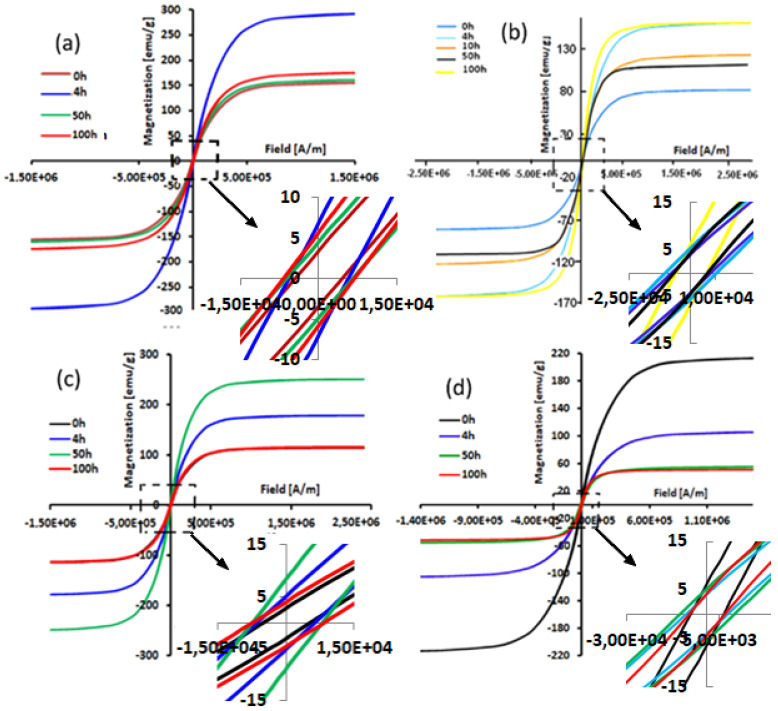
Typical hysteresis cycles (at 300 K) of the MA Fe_60−x_Co_25_Ni_15_Si_x_ alloys: x = 5 (**a**), x = 10 (**b**), x = 20 (**c**), and x = 30 (**d**) at.% for selected milling times. (Units: 1 emu/g = 1 Am^2^Kg). Notation: E+06 corresponds to × 10^6^, symbol—correspond to minus.

For the compound with 20% Si (Figure 3c), the substitution of cobalt in the iron matrix to form the bcc-Fe(Co) phase is identified after a milling time of 50 h. A good Rietveld refinement shows the coexistence of the Fe(Co) phase (Im-3m; a = 2.8675(1) Å; wt(%)~65) with the free elements cobalt (P63/mmc; a = 2.5057(1) Å and c = 4.0694(1) Å; wt(%)~15), nickel (Fm-3m; a = 3.5237(1) Å; wt(%)~13), and silicon (Fd-3m; a = 5.4293(2) Å; wt(%)~7). The same phases are also refined after 100 h of milling. For a sample with 30% Si, the formation of the Fe(Co) solid solution (Im-3m; a = 2.8692(1) Å; wt(%)~55) was observed after 2 h milling (Figure 3d). After 50 h of milling, Rietveld refinement was successful by three phases: Fe(Co,Si) (Im-3m; a = 2.8530(1) Å; wt(%)~62.5) and Fe(Ni) (Im-3m; a = 2.8670(1) Å; wt(%)~31) solid solutions with silicon (Fd-3m; a = 5.3670(1) Å; wt(%)~6.5) (Figure 4c)., while the hcp-Co has completely disappeared in the Fe lattice. At 100 h of milling, two metallurgical states were identified: an amorphous phase (proportion~65%) and a Fe(Co,Ni,Si) solid solution (Im-3m; a = 2.8526(1) Å; wt(%)~35).

Table 1 shows the microstructural parameters of the bcc-phase main phase as a function of milling time. The crystallite size of elemental Fe obtained before milling was about 150 nm. After the first 2 h of milling, the solid solution is formed and a rapid decrease of size (<80 nm) is observed. With continuing the milling up to 50 h, additional crystallite size refinement occurs slowly to a value of 33 nm in the samples with 5%Si and down to a value of 17 nm for the powder with 30%Si. After 100 h of milling, the refined average crystallite size was around 10 nm for the sample with 30%Si and 20 nm for the sample containing lower Si content.

In all compositions, the decrease of crystallite size is greater in the first step of mechanical alloying, while further milling slightly diminishes the crystallite size. This behavior can be related to the work-hardening stage which increases defects density (grains boundaries, dislocations, etc.). At the extended stages of the milling process, a remarkable transition from work-hardening to layered nanostructures regions may occur. When severe deformation takes place, the dislocation density tends towards a small critical value which may cause subsequent recrystallization, and for that, the size of the crystallite varies slowly. In addition, one can observe that the crystallite size of a powder with 30% Si appears smaller and thinner than other samples. This behavior hints that the solid solutions become harder with the addition of Si, which creates further fragmentation of the particles and, consequently, the obtention of small crystals [29]. In parallel, the microstrains increase as a function of milling time. This increase may be linked to the formation and movement of the dislocations [30,31,32]. By comparing all compositions, higher lattice strain values are obtained in the sample with 30% Si (Table 1). The difference in microstrain values were related to the Si segregation in grain boundaries, which made the growth of crystallites difficlut. Moreover, lattice strains in crystallites are generated by structure defects in terms of vacancies, dislocations, stacking faults, and grain boundaries. Generally, dislocations density, *ρ*s, can be represented in terms of the microstructural parameters, using the following equation cited in ref. [33]:

(1) $$\rho_{bcc}=\frac{2\sqrt{3}\times {\left(\varepsilon^{2}\right)}^{1/2}}{D\times b}$$

where ε is the lattice strain, D is the crystallite size, and the Burgers vector of dislocations, b, equals $\;a_{bcc}\frac{\sqrt{3}}{2}$ for the bcc structure. *a*_*bcc*_ is the lattice parameter fitted as a function of milling time for the bcc-structure. The calculated values of the dislocation densities, *ρ_bcc_*, as a function of milling time are given in Table 1. As shown, with the increase in milling time, the *ρ_bcc_* values increased for all sample compositions, and the highest value of 17.5 × 10^15^ m^−2^ is identified for the case of the sample doped with 30%Si and milled for 100 h. Moreover, one can note an increase in the dislocation density when the Si content increases. This may be due to the hardening and brittleness of the material by the diffusion of Si in the grain boundaries. The latter are used as barriers to dislocation movement, in the sense that when they turn hard or contract, the number of dislocations near the grain boundaries will increase.

### 3.3. Magnetic Properties

Figure 4 gives a superposition of hysteresis loops of the powder mixtures without Si and containing 5, 10, 20, and 30 at% Si as a function of selected milling time. All the milled powders had similar hysteresis loops indicating that these samples have a ferromagnetic behavior.

Further, all hysteresis cycles exhibited a sigmoidal shape with weak loss. This latter is characteristic of behavior in nanostructured samples with small magnetic domains [33]. Moreover, these low hysteresis losses are properties demanded in soft magnetic compounds [34]. As the milling time increases, the cycles become flattened. This may be due to the very high value of the magnetostriction coefficient of these alloys which is of the order of λ_s_ = 2.5 × 10^−6^ [35,36,37,38]. Moreover, the flattening of the hysteresis cycles of the powders when Si content increases can be explained by the decrease in coupling ferromagnetic.

**Figure 5:**
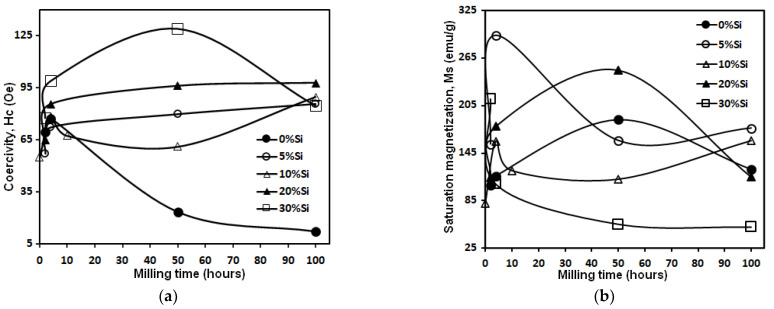
The evolution of the coercivity (Hc) (**a**) and the saturation magnetization (Ms) (**b**) of the MA Fe_60−x_Co_25_Ni_15_Si_x_ (x = 0, 5, 10, 20, and 30 at%Si) alloys on the milling time. (Units: 1 Oe = 10^−4^ T).

The dependence of the coercivity (Hc) and the saturation magnetization (Ms) on the milling time are given in Figure 5. All mechanically alloyed powders have a soft magnetic behavior (Hc ≤ 100 Oe) (Figure 5a). However, before 2 h of milling, an increase in Hc as a function of milling time and Si content additions of about 30 Oe is observed. This increase reflects the decrease in the coupling between ferromagnetic grains via the intergranular zone [39,40]. Based on the fact that the Fe, Co, and Ni alloys are ferromagnetic, the substitution of one of these elements by Si, which is not magnetic, leads to a decrease in ferromagnetic coupling [41]. However, it has been reported that dislocation density is the most important factor that affects coercivity [42]. Further, Yu et al. have observed experimentally that the increase of Hc is directly linked to the presence of grain boundaries, precipitates, and disordered phases [43]. Another cause of increased Hc may be surface anisotropy [43,44,45,46]. This latter may have origins in the interactions of exchange between the spins of the core and surface atoms [44,45,46,47]. Indeed, by reducing the particle size down to the nanometric scale, the surface/volume ratio increases, and hence the influence of surface anisotropy increases [48,49]. Then again, for the FeCoNi alloy without Si, we can notice a decrease in Hc up to a value of 12.4 Oe at 100 h of milling. In addition, a slight decrease of 15 Oe for the sample with 10% Si between 4 and 50 h of milling is observed. The reduction of Hc can be attributed to the reduction of magnetic anisotropy. The start of milling is characterized by a decrease in the volume fraction of hcp-Co, hence a reduction in anisotropy magneto-crystalline.

We recall that the hcp structures have a magneto-crystalline anisotropy higher than cubic structures [50]. In addition, the decrease in the coercive field, Hc, can also be related to the increase in the average particle size at the start of milling (by agglomeration). The particle size of powder results from the competition between the repeated phenomena of fracture and welding. With increasing particle size, the alloy becomes multi-domain, and the magnetization is done by displacement of walls. The magnetic softness is dependent on the reduction of residual tensions when the particle size increases [51,52].

The dependence of saturation magnetization (Ms) on the milling time can provide additional information on the magnetic behavior during the MA process. As can be seen in Figure 5b, Ms strongly depends on the chemical composition of the alloys. After 4 h of milling, the Ms decrease (for samples with 5, 10, 30%Si content) can be explained by the segregation of Si in the FeCoNi lattice. After 100 h, the saturation magnetization takes very close values, while for the samples without Si and with 20%, one can observe an increase after 4 h followed by a decrease after 50 h of milling. The inhomogeneous evolution of Ms as a function of Si content can be linked to the magnetostriction effective anisotropy and stress introduced during the milling process. As a result, one can deduce that Ms is independent of the microstructure and strongly sensitive to the chemical composition; the opposite of coercivity Hc, Ms is structure insensitive.

## 4. Conclusions

Nanostructured Fe_60−x_Co_25_Ni_15_Si_x_ (x = 0, 5, 10, 20, and 30 at%) alloys were prepared by a high-energy mechanical ball milling. Microstructural and magnetic properties as a function of milling time were investigated. The formation of the supersaturated Fe(Co,Ni,Si) solid solution was identified by the use of XRD diffractograms after 100 h of milling. The increase in the Si content favors the work hardening of powdered alloys resulting in particle and crystallite size reduction and lattice strain rise. Increasing the milling time and adding Si into Fe, Co, and Ni mixture metals at the same time diminishes the Ms due to the microstructure refinement and Si diamagnetic effect. The final mechanically alloying products showed a soft magnetic behavior (Hc ≤ 100 Oe).

## Figures and Tables

**Table 1:** Refined average crystallite sizes, <D> (±5 nm), lattice strains, ε (±0.02%), and dislocation density, *ρ_bcc_* (±0.05 × 10^15^ m^−2^), for Fe_60−x_Co_25_Ni_15_Si_x_ powders (x = 0, 5, 10, 20, and 30 at.%).

t(h)	Fe_55_Co_25_Ni_15_Si_5_			Fe_50_Co_25_Ni_15_Si_10_			Fe_40_Co_25_Ni_15_Si_20_			Fe_30_Co_25_Ni_15_Si_30_		
	<D> (nm)	ε (%)	*ρ_bcc_* (m^−2^) ×10^15^	<D> (nm)	ε (%)	*ρ_bcc_* (m^−2^) ×10^15^	<D> (nm)	ε (%)	*ρ_bcc_* (m^−2^) ×10^15^	<D> nm	ε (%)	*ρ_bcc_* (m^−2^) ×10^15^
0	150	0.005	0.046	150	0.005	0.046	150	0.005	0.046	150	0.005	0.046
2	74	0.12	0.16	75	0.14	0.16	78	0.18	0.23	80	0.25	0.26
4	65	0.25	0.48	70	0.15	0.27	75	0.20	0.35	76	0.35	0.41
10	55	0.29	0.73	50	0.38	1.05	47	0.45	1.3	38	0.5	1.8
25	41	0.35	1.1	38	0.65	2.3	35	0.55	2.1	25	0.65	3.3
50	33	0.88	3.7	30	0.91	4.2	25	0.98	5.4	17	1.05	9.8
80	27	0.87	4.4	25	0.95	5.3	20	1.02	6.9	12	1.20	14.07
100	20	0.95	5.3	17	1.05	7.3	15	1.1	8.5	10	1.25	17.5

## Data Availability

Data can be requested to the authors.
